# Mental health and governmental response policy evaluation on COVID-19 based on vaccination status in Republic of Korea

**DOI:** 10.1186/s12889-023-16514-w

**Published:** 2023-08-25

**Authors:** Dong-Kyu Kim, Inyup Lee, Chulhwan Choi, Sung-Un Park

**Affiliations:** 1 Department of Sports Science, Chungwoon University, Chungcheongnam-Do Hongseong-gun, Republic of Korea; 2 Department of Sports Medicine, Kyunghee University, Yongin-si, Gyeonggi-do Republic of Korea; 3 Department of Physical Education, Gachon University, Seongnam-si, Gyeonggi-do Republic of Korea; 4 Department of Sports and Health, Hwasung Medi-Science University, Hwasung-si, Gyeonggi-do Republic of Korea

**Keywords:** Vaccination, COVID-19, Mental health, Quarantine, Exercise

## Abstract

**Background:**

The coronavirus disease (COVID-19) pandemic has continued since the outbreak in December 2019. People experience depression and anxiety due to government policies and restrictions on physical activity due to the COVID-19 pandemic. This study aimed to compare and analyze people’s experiences of COVID-19 blues, sports policy awareness, and participation intention according to their vaccination status.

**Methods:**

This quantitative study used an online survey to collect demographic information, vaccination status, and variables. Data validity and reliability were verified through confirmatory factor analysis, and Cronbach’s alpha coefficients were calculated using SPSS/AMOS 23.0. Finally, this comparative study was conducted using multivariate analysis of variance to investigate the differences in the dependent variables between the groups.

**Results:**

The vaccinated group had higher scores for all factors related to COVID-19 blues (*F* = 19.147; *p* < .05; partial *η*^*2*^ = .046) and government policy (market responsiveness: *F* = 5.669, *p* < .05, partial *η*^*2*^ = .014; policy performance: *F* = 6.997, *p* < .05, partial *η*^*2*^ = .017; policy satisfaction: *F* = 7.647, *p* < .05, partial *η*^*2*^ = .019), apart from the intention to participate in sports (*F* = .014, *p* > .05, partial *η*^*2*^ = .000); these results demonstrate that people with COVID-19 blues and relatively high confidence in government quarantine policies were more likely to be vaccinated. In addition, all participants gave sports-participation intention the highest rating, regardless of their vaccination status; this reflects the current situation, in which individual activities are limited.

**Conclusions:**

This study analyzed the mental health of vaccinated and unvaccinated groups in Korean adult men, their perceptions of government policies, and their willingness to engage in physical activity. The findings are meaningful and highlight useful directions for future research. This study provides evidence which can help alleviate the mental damage caused by government quarantine policies and enable a better understanding of the COVID-19 pandemic.

The results of this study provide important data for understanding the COVID-19 pandemic.

## Introduction

People are suffering the consequences of the ongoing coronavirus disease (COVID-19) pandemic, which began at the end of 2019 [1]. The spread of infection through contact with people and droplets has resulted in halting industrial operations and impeding human relationships. The rapid spread and fatality rate of COVID-19 are threatening and not comparable to previous outbreaks of Middle East Respiratory Syndrome and Severe Acute Respiratory Syndrome [2]. In the subsequent phase, vaccine development and rapid inoculation raised the hope that people would be free from the virus. Governments in all countries have made continuous efforts to limit the spread of COVID-19 by encouraging vaccination and mobilizing policies that provide economic benefits to those who have completed vaccination [3]. However, despite these efforts, as new strains of COVID-19 develop, the effectiveness of the vaccine is uncertain [4], resulting in the paralysis of many nations’ industries and people’s daily lives.

Most people are afraid of the virus and follow their government’s changing quarantine rules framed on the basis of the daily number of confirmed cases and deaths. The government’s quarantine rules encourage vaccination. They focus on minimizing contact between people and compensating for resulting losses [5]. However, mutant COVID-19 strains have resulted in public fatigue and dissatisfaction related to the ongoing pandemic due to frequent changes in quarantine policies and additional booster shots [4]. In addition, when vaccinations are incomplete, people feel restricted in their daily lives [6]. In Korea, “vaccinated person” generally refers to someone who has completed a second vaccination less than 180 days ago. In contrast, even if a secondary vaccination has not been completed or 180 days have passed, the person is classified as unvaccinated and faces restrictions in daily life.8 Eventually, the government introduced a policy called the “vaccine pass,” which is issued through smartphones only to those who have completed vaccination, based on data from the Korea Centers for Disease Control and Prevention [7]. This pass serves as a vaccine-related identification card [8]. It is also a point of conflict between the government’s efforts to cope with the virus – that change daily – and the interests of the people who have to live with the restrictions of daily life.

Despite the government’s policy efforts and people’s compliance with quarantine policies, depression, due to the current situation of uncertainty and prolonged disconnections in daily life caused by the threat of COVID-19, is on the rise [9, 10]. Depression caused by the prolonged pandemic is referred to as COVID-19 blues [11]. This term is a combination of the words COVID-19 and blues, meaning “depression and anxiety from COVID-19.” Another term used is Corona Blue [12]. The symptoms of COVID-19 blues can often be identical to those of depression or anxiety, but the difference is in their duration [13]. In addition, if one is consistently distracted from normal life routines, such as work or home life, it could be a sign of a more serious COVID-19 problem [13]. Therefore, the government recommends physical activity to alleviate direct viral infections and COVID-19 blues [14, 15]. It suggests activities that can be performed at home, such as home training and outdoor activities with a limited number of people, but not in a closed place where many people gather. Several studies have shown that physical activity can benefit people physically, mentally, and socially [16–18]. It is essential to engage in physical or social activities that comply with the government quarantine rules.

Given that the emergence of new COVID-19 strains has prolonged the pandemic, vaccination is required to overcome COVID-19; thus, regulations on the lives of unvaccinated people are increasing. This situation implies that the gap between vaccinated and unvaccinated individuals in daily life may continue to widen. Therefore, in this study, the presence or absence of vaccination was taken as the key critical factor affecting vaccination status. The study participants were subdivided by their vaccination status. Three factors, (a) the COVID-19 blues experience, (b) perceptions of government policy, and (c) intention to participate in physical activities were compared and analyzed. To date, no studies have compared and analyzed the current situation by setting vaccination status as a major factor. The results of this study can be applied to future government decisions related to COVID-19 policy and provide meaningful data to explore how individuals who are not infected with the virus perceive the COVID-19 outbreak.

## Materials and Methods

### Data collection procedure

Data collection was implemented via the NAVER (NAVER Corp., Seongnam, Republic of Korea) online survey platform for two months, starting in November 2021. Adults over 20 years of age who enjoyed various sports participated in the survey. Data were distributed and collected only from participants who voluntarily agreed to participate after the research purpose, and the fact that there were no benefits or disadvantages to participating in the survey was explained. This study investigated the psychological characteristics of COVID-19 blues, market responsiveness of sports-related policy, policy performance, policy satisfaction, and participation intention in sports; therefore, minors were excluded. Finally, this study did not collect sensitive personal information from the respondents.

### Instruments

To measure COVID-19 blues in the context of the COVID-19 pandemic, a factor including five items (e.g., “I feel hypersensitive and nervous because of COVID-19”) developed by Lee [19] was applied. A factor including three items (e.g., “during the COVID-19 pandemic, sports policies were properly announced when necessary”) developed in a previous study was utilized to investigate the market responsiveness of sports-related policy. Another factor including four items (e.g., “sports policies before COVID-19 have achieved results in increasing participation in sports”) used in previous research [20, 21], was adopted in this study to estimate sports-related policy performance during the COVID-19 pandemic. A third factor with four items (e.g., “During the COVID-19 pandemic, I am satisfied with the current government’s fair sports-related policies compared to that of other fields”) used in a study by Shin and Choi,20 was supplemented and applied in this study to measure sports-related policy satisfaction. Finally, to investigate participation intention in sports, a factor including four items (e.g., “I will participate in sports regardless of the surrounding situation”) developed by Kim and Ko [22] was modified and applied.

### Data analysis

All statistical analyses were conducted using the SPSS Windows software version 23 and AMOS 23.0. First, this analysis showed the sociodemographic data (e.g., sex, age, preferred sports type, and frequency of participation in sports) of the study respondents. Second, to verify the scale validity of this study, a confirmatory factor analysis (CFA) was performed on five factors: COVID-19 blues, market responsiveness, policy performance, policy satisfaction, and participation intention in sports. Cronbach’s alpha was used to test the reliability of the data. Finally, multivariate analysis of variance (MANOVA) was conducted to investigate the differences in the dependent variables between the two groups segmented by an independent variable (i.e., vaccination status).

## Results

### Study participants

A total of 550 questionnaires were distributed online, and 399 responses were received (approximately 72.55% response rate) after automatically excluding 151 incomplete responses from the online platform. Survey respondents were categorized into two groups depending on their vaccination status, which was applied as an independent variable (Group 1: vaccinated; Group 2: unvaccinated). In this study, the term “vaccinated” refers to completing the second dose of the COVID-19 vaccine. In contrast, the term “unvaccinated” refers to those who were vaccinated once or never. Furthermore, all survey respondents provided demographic information, including sex, age, preferred sport type, and frequency of participation in sports. Table 1 shows the results of the detailed descriptive statistical analysis of survey respondents.

**Table 1:** Descriptive statistic

**Items**		**Group 1 (Vaccinated)**	**Group 2 (Unvaccinated)**
Gender	Male	117 (56.5%)	105 (54.7%)
	Female	90 (43.5%)	87 (45.3%)
Age	20 s	60 (30.9%)	104 (54.2%)
	30 s	35 (16.9%)	29 (15.1%)
	40 s	36 (17.4%)	38 (19.8%)
	50 s and over	76 (36.7%)	21 (10.9%)
Preferred sports type	Indoor	89 (43.0%)	105 (54.7%)
	Outdoor	118 (57.0%)	87 (45.3%)
Participation in sports(per week)	Less than one day	64 (30.9%)	47 (24.5%)
	1–2 days	75 (36.2%)	74 (38.5%)
	3–4 days	45 (21.7%)	47 (24.5%)
	More than 5 days	23 (11.1%)	24 (12.5%)
Total		207 (51.88%)	192 (48.12%)

### Scale validity and reliability

A confirmatory factor analysis was conducted to verify the validity of the research tool used. All values (factor loading, average variance extracted, and construct reliability) exceeded the statistical standards. Additionally, the goodness-of-fit test revealed satisfactory results for all statistical baselines (CMIN = 432.824, DF = 160, CMIN/DF = 2.705, NFI = 0.943, CFI = 0.963, RMSEA = 0.065, RMR = 0.037).

In addition, the reliability between items of the factors was tested with the value of Cronbach’s alpha, applying a satisfactory statistical cutoff value of 0.70 [23], yielding the values COVID-19 blues, α = 0.942, market responsiveness, α = 0.919, policy performance, α = 0.922, policy satisfaction, α = 0.973, and participation intention in sports, α = 0.925. Thus, the instruments used in this study had satisfactory statistical reliability. The scale validity and reliability results are presented in Table 2.

**Table 2:** Confirmatory factor analysis and reliability analysis

**Construct and scale items**	***λ***	**AVE**	**C.R**	***α***
**COVID-19 blues**				
I feel depressed because of COVID-19.	.828	.767	.943	.942
I am annoyed by small things because of COVID-19.	.897			
I feel hypersensitive and nervous because of COVID-19.	.907			
I spend most of my time depressed by COVID-19.	.890			
I feel helpless for a lot of time because of COVID-19.	.853			
**Market responsiveness**				
During the COVID-19 pandemic, sports policies were properly announced when necessary.	.861	.795	.921	.919
During the COVID-19 pandemic, sports policies were announced.	.917			
During the COVID-19 pandemic, sports policies comprehensively considered sports institutions and sports participants.	.896			
**Policy performance**				
Sports policies before COVID-19 were effective for public health.	.861	.747	.922	.922
Sports policies before COVID-19 were for the people.	.843			
Sports policies before COVID-19 contributed to the sports market.	.899			
Sports policies before COVID-19 have achieved results in increasing participation in sports.	.853			
**Policy satisfaction**				
During the COVID-19 pandemic, I am satisfied with the current government’s fair sports-related policies compared to that of other fields.	.911	.808	.944	.973
During the COVID-19 pandemic, I am satisfied with the current government’s responsible sports policies.	.892			
During the COVID-19 pandemic, I am satisfied with the current government’s consistent sports policies.	.887			
During the COVID-19 pandemic, I am generally satisfied with the current government’s sports policies.	.904			
**Participation intention in sports**				
I will participate in sports regardless of the situation.	.721	.771	.930	.925
I am willing to participate in sports in the future.	.921			
I have a plan to participate in sports in the future.	.969			
I will try to participate in sports in the future.	.881			

CMIN = 432.824, DF = 160, CMIN/DF = 2.705, NFI = .943, CFI = .963, RMSEA = .065, RMR = .037

### Multivariate analysis of variance (MANOVA)

A MANOVA for the comparative study was conducted to determine the differences in COVID-19 blues, market responsiveness, policy performance, policy satisfaction, and participation intention in sports by vaccination status during the COVID-19 pandemic. First, homogeneity of covariance was verified (Box’s M = 60.485, *F* = 3.978, *p* < 0.001). Next, statistically significant differences between the groups were revealed (Wilks’ lambda = 0.940, *F* = 5.015, *p* < 0.001). As reported in Table 3, statistically significant differences between groups were found in four factors: (a) COVID-19 blues, (b) market responsiveness, (c) policy performance, and (d) policy satisfaction. In contrast, statistically significant differences were not found for participation intention in sports. The mean scores of all dependent variables in the groups (vaccinated and unvaccinated) are shown in Table 3. Specifically, the mean scores of all depend.

**Table 3:** Results of multivariate analysis of variance

**Dependent Variables**	***df***	**F**	***p***	***η***^***2***^	**Mean (SD)**	
					**Group 1**	**Group 2**
COVID-19 blues	1	19.147	.000^***^	.046	3.033 (.995)	2.587 (1.043)
Market responsiveness	1	5.669	.018^*^	.014	2.821 (.901)	2.601 (.950)
Policy performance	1	6.997	.008^**^	.017	3.271 (.860)	3.030 (.956)
Policy satisfaction	1	7.647	.006^**^	.019	2.714 (.931)	2.473 (.799)
Participation intention	1	.014	.904	.000	3.953 (.757)	3.944 (.737)

*Group 1* Vaccinated, *Group 2* Unvaccinated

^*^p < .05

^**^p < .01

^***^p < .001

## Discussion

The spread of COVID-19, which has paralyzed people’s daily lives and industrial and economic activities worldwide, has been ongoing for over two years since the end of 2019. The fear experienced at the time of the virus outbreak has gradually disappeared with the development of vaccines and subsequent inoculation. Moreover, national governments are attempting to overcome the virus through the quarantine rules they have implemented. However, despite these efforts, COVID-19 has had a tremendous impact on daily life and economic activities, and people are emotionally exhausted. It is true that people feel depressed and have doubts about quarantine policies. Additionally, they are eager to participate freely in physical activities that provide physical, mental, and social benefits. Vaccination depends on individuals’ free will. It is essential to compare and analyze perceptions regarding COVID-19 blues, quarantine policy, and physical activity by vaccination status in the current situation, which is characterized by encouraging vaccination and expanding regulations on unvaccinated people.

First, in this study, the analysis results for defined depression, COVID-19 blues [19], which is defined as depression caused by a prolonged pandemic, showed high results in the group that had completed vaccination, contrary to expectations. More precisely, this result is the opposite of what was generally expected since this study assumed that unvaccinated people would experience depression due to fear of viral infection and restrictions on their daily lives. However, as mentioned earlier, vaccination can be determined by individual freedom; therefore, this study can easily determine whether an individual’s free will is higher than the fear of virus infection as a group. Results find that people with relatively low psychological pressure related to the virus (e.g., depression and fear) may be interpreted as passive regarding vaccination. This study thus conclude that these personal tendencies and beliefs have caused statistically significant differences between the groups regarding COVID-19 blues. Thus, COVID-19 blues is emerging as a social issue because of prolonged virus outbreaks. Increasing vaccine stability and resistance to vaccination seems to be a more effective policy to overcome COVID-19 than quarantine policies that enforce the fear of infection or restrictions in daily life.

Second, a comparative analysis of the perceptions of the government’s quarantine policy on COVID-19 according to vaccination status revealed that the vaccinated group showed relatively higher scores than the unvaccinated group in all three sub-factors: market response, policy performance, and policy satisfaction. This can be easily understood from the perspective that vaccination is the most important policy among government quarantine policies. More specifically, because individuals can make their own decisions regarding vaccination, it can be argued that those who have completed vaccination will accept the government’s quarantine policy. The government’s quarantine policy has continued to change, while the pandemic has been prolonged due to the outbreak of mutant viruses (e.g., the delta and omicron variants), resulting in public dissatisfaction. Even in this situation, it is expected that a high evaluation of quarantine policy was derived from the group that complied with the government’s quarantine policy and quickly completed vaccination to overcome the virus. Unfortunately, the experience of COVID-19 blues is relatively high, despite active compliance with quarantine policies. Considering these results, this study acknowledges the importance of effective quarantine policies. The government should show that compliance with quarantine policies can enhance their physical, psychological, and social health during the COVID-19 pandemic.

Finally, physical activity was found to be considered the best method for individuals to implement, independent of medical policies to overcome COVID-19, and is one of the important factors in the present study. This can be seen in prior findings that the various benefits of physical activity identified in many previous studies [16, 24, 25] are another realistic and useful level of quarantine policy for maintaining individual health. Continuous physical activity can be argued to be the best choice that an individual can make to the extent that it complies with the government’s quarantine rules. There was no statistically significant difference in the intention to participate in physical activity based on vaccination status in this study. However, this is considered a remarkable result because it showed the highest mean score among all the factors. Regardless of individual judgment, perception of vaccination, and the government’s quarantine policy, it can be interpreted that all individuals had a high level of desire for physical activity. As revealed in previous studies, the results of this study can be considered another confirmation of the countless benefits of physical activity. Therefore, physical activity can play a role in overcoming factors that harm psychological health, such as COVID-19 blues, as part of an effective quarantine policy.

### Limitations

This study compared and analyzed the psychological health of vaccinated and unvaccinated groups, their perceptions of government policies, and their willingness to engage in physical activity. The results of this study yielded meaningful results, and they highlight directions for future research.

First, this study conducted an analysis by dividing vaccinated and unvaccinated people based on the premise that there would be differences in their perceptions of government quarantine policies and vaccinations. This assumption was possible because the government’s long-term recommendations for vaccination had already been publicly issued and the possibility of non-vaccination due to external factors was very low. However, this is a research limitation because there may be people who have not been vaccinated for personal reasons, such as health conditions, and not due to external factors. Therefore, to conduct a comparative study by vaccination status in the future, an additional question regarding the reason for remaining unvaccinated is required.

Second, there are practical research limitations to the government’s quarantine policy, which is an important factor in this study. The government’s quarantine policy has undergone numerous changes during the COVID-19 pandemic. Therefore, people’s perceptions of quarantine policies might have changed substantially over time. Additional research on quarantine policies should analyze changes in people’s perceptions over time. This is an effective research approach for identifying objective changes in perceptions. Furthermore, significant factors, such as vaccine development and mutant virus outbreaks, should be considered time-based. Third, various external factors such as fear, uncertainty, and social isolation could affect individuals’ mental health during the pandemic. These factors can influence people’s perceptions, attitudes, and behaviors, including their willingness to engage in PA. Future studies should incorporate these external factors to enhance the robustness and applicability of our findings.

## Conclusions

This study analyzed the mental health of vaccinated and unvaccinated adult Korean men, their perceptions of government policies, and their willingness to engage in physical activity. These findings are significant and highlight valuable directions for future research. This study provides evidence to help alleviate the mental damage caused by government quarantine policies and enable a better understanding of the COVID-19 pandemic. These findings can serve as valuable primary data for government policy establishment, responding to the current pandemic and preparing for potential future national risks. Understanding the factors influencing mental health, perceptions, and behaviors during a public health crisis can inform the development of targeted interventions, support systems, and policy strategies. With this knowledge, governments can better address the mental health consequences of quarantine policies, promote well-being, and improve overall crisis management. The results of this study provide essential data for evidence-based decision-making and contribute to building a resilient and responsive healthcare system.

## Data Availability

The datasets used and/or analysed during the current study available from the corresponding author on reasonable request.

## Abbreviations

AVE: Average Variance Extracted
CFI: Comparative Fit Index
CMIN: Comparative Fit Index
COVID-19: Coronavirus Disease 2019
C.R.: Composite Reliability
***λ***: Factor loadings
***α***: Cronbach’s alpha
DF: Degrees of Freedom
MANOVA: Multivariate Analysis of Variance
NFI: Normed Fit Index
RMR: Root Mean Square Residual
RMSEA: Root Mean Square Error of Approximation
